# Ligand-Mediated Proteome Remodeling Shapes Nanoparticle Protein Corona Composition for Deep Plasma Profiling

**DOI:** 10.21203/rs.3.rs-9421288/v1

**Published:** 2026-04-28

**Authors:** Bahareh Ghaffari, Liuchenxin Han, Ali Tamadon, Alphan Alpaydin, Ghafar Yerima, Nick Wills, Shaun Grumelot, Danilo Ritz, Alexander Schmidt, Sylvain Peuget, Mohammad Ghassemi, Hojatollah Vali, Mohammad R K Mofrad, Amir Ata Saei, Babak Borhan, Morteza Mahmoudi

**Affiliations:** 1 Department of Chemistry, Michigan State University, East Lansing, Michigan 48824, USA; 2 Precision Health Program, Michigan State University, East Lansing, Michigan 48823, USA; 3 Department of Microbiology, Tumor and Cell Biology, Karolinska Institute, Stockholm 171 77, Sweden; 4 Molecular Cell Biomechanics Laboratory, Departments of Bioengineering and Mechanical Engineering, University of California Berkeley, Berkeley, CA, USA.; 5 Department of Radiology, College of Human Medicine, Michigan State University, East Lansing, Michigan 48823, USA; 6 Proteomics Core Facility, Biozentrum, University of Basel, Basel, Switzerland; 7 Department of Computer Science and Engineering, Michigan State University, East Lansing, Michigan 48824, USA; 8 Department of Anatomy and Cell Biology, McGill University, Montreal, QC, H3A 0C7, Canada; 9 Facility for Electron Microscopy Research, McGill University, Montreal, QC, H3A 0C7, Canada

## Abstract

The human plasma proteome contains extensive diagnostic information but remains difficult to interrogate because protein concentrations span more than ten orders of magnitude, with highly abundant proteins such as albumin masking low-abundance biomarkers. Nanoparticle (NP) enrichment strategies partially address this limitation, but their analytical depth is still constrained the incorporation of albumin and other highly abundant proteins within the protein corona. Here we show that the NP protein corona can be rationally reprogrammed by pre-incubating plasma with a chemically diverse panel of ligands designed to bind albumin, attenuate its interaction with NPs, and promote enrichment of lower-abundance proteins. Molecular docking and structural analyses demonstrate that these ligands induce allosteric conformational changes and surface charge redistribution on albumin, thereby reducing its association with the NP surface. Importantly, this strategy is highly tunable, as individual ligands shift protein corona composition toward different broad families of proteins, revealing overall enrichment trends rather than strict selectivity for any single class. Using this approach, we quantified over 6,600 proteins from a single human plasma sample across a curated panel of conditions, substantially exceeding the depth achieved with untreated NP enrichment. These findings establish a versatile chem–bio strategy for ligand-directed protein corona engineering, enabling high-depth plasma proteome profiling and expanding access to low-abundance proteins and candidate biomarkers across human diseases.

The human plasma proteome is a vital reservoir of biological information, containing protein signatures that reflect nearly every physiological and pathological state.^1^ As such, it remains a cornerstone for biomarker discovery and the advancement of personalized medicine.^1, 2, 3^ However, the full potential of plasma proteomics is fundamentally constrained by the exceptionally wide dynamic range of protein concentrations. Specifically, highly abundant proteins such as albumin overshadow low-abundance biomarkers that may be crucial for early disease detection and personalized medicine.^4, 5, 6^ Consequently, overcoming this “albumin bottleneck” to achieve deeper proteome coverage remains a primary objective in clinical proteomics. Nanoparticles (NPs) have emerged as a transformative tool for addressing this challenge because they can reduce the complexity of the plasma proteome through their unique physicochemical properties, most notably their high surface area-to-volume ratio and customizable surface chemistry.^7, 8, 9^

Upon exposure to biological fluids, NPs rapidly adsorb diverse plasma proteins, forming a “protein corona” that is representative of the biological fluid.^10, 11, 12, 13^ This corona formation can help overcome the challenges posed by the plasma proteome,^10^ by preferentially capturing and enriching a broader spectrum of proteins, including those present at low concentrations, thereby enhancing proteome coverage and facilitating improved detection and analysis of potential biomarkers.^7, 8^ Despite this enrichment capability, albumin and other highly abundant proteins still constitute a significant portion of the protein corona.^14^ Crucially, conventional affinity depletion techniques lack tunability and are costly and labor intensive for large cohorts.^15^ Therefore, the central challenge is not merely the removal of highly abundant proteins such as albumin, but the controlled remodeling of proteome–NP interactions in a manner that preserves the underlying proteomic landscape while enhancing access to lower-abundance proteins. Herein, we demonstrate that small-molecule interactions with the plasma proteome, including albumin, reprogram protein incorporation into the NP corona. This strategy enhances the binding and detection of lower-abundance proteins and broadens representation across protein families, enabling deeper proteome analysis while maintaining the integrity of the captured proteome.

To reduce albumin incorporation and expand the dynamic range of proteins accessible by mass spectrometry, two major strategies have been proposed. The first relies on panels of multiple NPs with distinct physicochemical properties, each of which enriches different subsets of lower-abundance proteins, thereby increasing overall proteome coverage.^7, 16^ This approach leverages the strong dependence of protein corona composition on NP characteristics. The second strategy relies on spiking plasma with small organic molecules (e.g., lipids, metabolites, and synthetically designed ligands) that can not only attenuate the association of highly abundant proteins with NPs, but also enhance the association of low-abundance protein families, thereby reshaping the structure of the protein corona.^47^ This latter approach enables deep plasma proteome profiling using a single NP formulation, offering significant advantages in terms of assay scalability, analytical reproducibility, and minimal sample volume requirements.

Building on the concept of introducing exogenous small molecules, we present a systematic strategy to test whether targeting albumin with high-affinity ligands can enable enrichment of proteins not observed in the untreated NP corona. We selected a chemically diverse panel of small molecules, including drugs, fatty acids (FAs), and synthetic ligands, designed to engage specific functional domains of albumin. Our results suggest that their effects are not explained solely by reduced albumin incorporation into the corona. Rather, ligand binding appears to reshape albumin-associated interactions within the plasma proteome. By selectively occupying these sites, these molecules may induce allosteric and electrostatic changes that perturb albumin–protein interactions and render additional low-abundance proteins accessible for NP capture. This reorganization of corona assembly increases the NP’s capacity to recruit a broader and more diverse set of low-abundance proteins (Figure 1A). Ultimately, our findings demonstrate that targeted molecular interference can expand proteome depth and alter corona composition beyond that achieved in the untreated condition, providing a high-depth analytical platform for biomarker discovery and translational diagnostics.

## Mechanistic selection of small molecules for albumin engagement

**Figure 1.**
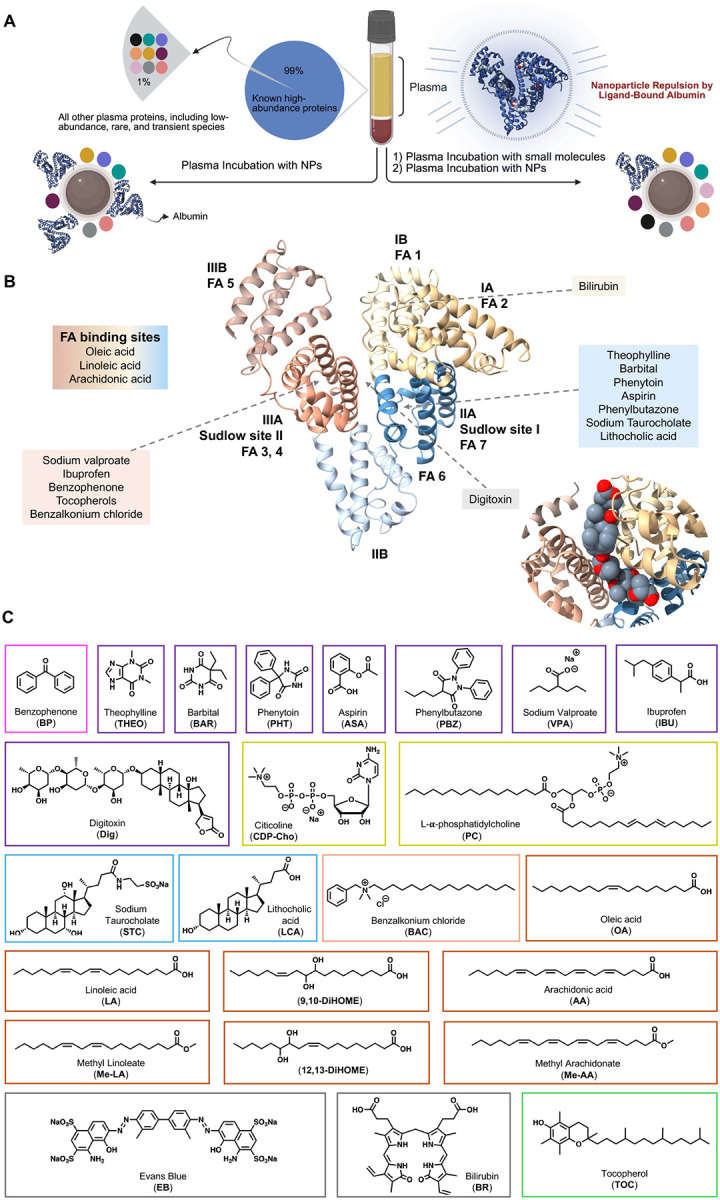
Small-molecule modulation of albumin adsorption within the NP protein corona. (A) Conceptual overview of the experimental approach in which plasma is pre-incubated with albumin-binding small molecules prior to NP exposure, with the aim of reducing albumin dominance and enhancing the depth and coverage of proteins recruited to the NP corona. (B) Structural representation of HSA highlighting major ligand-binding regions, including FA binding sites and Sudlow sites I and II, illustrating how ligands from distinct molecular classes engage different albumin pockets. Inset: zoomed-in view of the docked pose of digitoxin (cyan) bound at the IA/IIIA interface of human serum albumin b. (C) Chemical structures of representative small molecules spanning diverse ligand classes including drugs, FAs, bile acids, phospholipids, nucleotides, and synthetic and endogenous pigments used in this study to systematically probe albumin–NP interactions. Chemical structures of ligands are highlighted with colored boxes: purple (drugs), light blue (bile acids), green (vitamins), orange (surfactant), and red orange (fatty acids and derivatives), gray (endogenous and synthesized dyes), yellow (phospholipids/nucleotides), and magenta (aromatic hydrophobic compounds).

Since the initial elucidation of the three-dimensional structure of human serum albumin (HSA) in 1989,^17^ extensive research has detailed its sophisticated ligand-binding architecture.^18, 19, 20, 21, 22^ HSA is composed of three homologous domains (I-III), each subdivided into two distinct subdomains, A and B. Central to its transport function are two high-affinity ligand-binding regions: Sudlow site I, located in subdomain IIA, and Sudlow site II, situated in subdomain IIIA (Figure 1B). These pockets exhibit distinct chemical microenvironments: binding at Sudlow site I is primarily governed by hydrophobic interactions within a large, flexible interior, whereas Sudlow site II relies on a complex synergy of hydrogen bonding, electrostatic forces, and hydrophobic contacts within a more constrained space. Additionally, subdomain IB has emerged as a primary or secondary docking site for a variety of clinical drugs. Together, these structural features define a versatile ligand-binding landscape that can be strategically exploited to regulate albumin behavior at NP interfaces.

**Figure 2.**
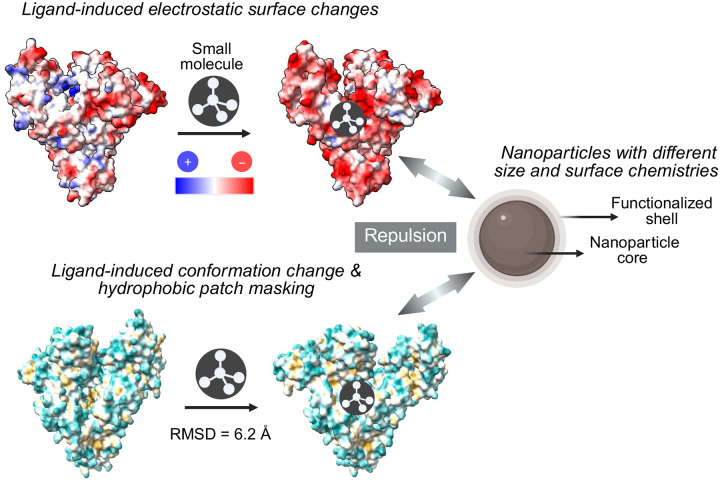
Schematic illustration of ligand-induced remodeling of albumin to modulate albumin–NP interactions and protein corona composition. The top panel shows HSA before and after binding phenylbutazone (PBZ), illustrating a pronounced redistribution of surface electrostatic potential toward a more negative profile upon ligand binding. The bottom panel shows HSA before and after binding oleic acid, highlighting a substantial ligand-induced conformational rearrangement with a backbone RMSD of 6.2 Å. PDB IDs: HSA apo (1ao6), HSA bound PBZ (2bxc), HSA bound oleic acid (1gni).

Beyond drug-binding sites, HSA possesses multiple sites with varying affinities for long-chain FAs, typically characterized by hydrophobic pockets capped with polar or charged amino acids.^18, 23, 24, 25^ These well-defined structural features provide a strategic roadmap for curating a targeted panel of ligands to modulate HSA–NP interactions. Specifically, we selected the specific suite of small molecules presented in Figure 1C to operate via two primary mechanisms: (1) direct steric occlusion, in which ligands bind with high affinity to occlude specific surface-exposed pockets, thereby physically preventing their interaction with the NP surface; and (2) allosteric remodeling, in which ligands engage internal sites to trigger global conformational rearrangements or reorient surface residues. This redistribution of surface electrostatics effectively disfavors NP association through increased repulsion or reduced affinity (Figure 2).

To block albumin–NP interactions, we assembled a chemically and structurally diverse panel of albumin-binding molecules encompassing hydrophobic and heterocyclic compounds, FAs, phospholipids, surfactants, nucleotides, and both synthetic and endogenous pigments (Table 1). These ligands span a wide range of molecular weights, chemical scaffolds, and physicochemical properties, and collectively engage distinct binding sites on albumin, enabling systematic remodeling of its surface characteristics (Figure 1B, C).

## Results and Discussion

### Docking and molecular dynamics simulations predict the ligand’s binding sites on albumin

Molecular docking and molecular dynamics (MD) simulations were performed to investigate interactions between HSA and selected phosphatidylcholine-related small molecules (Table S1). Building on our previous report showing that L-α-phosphatidylcholine modulates protein corona composition,^47^ we screened a series of related ligands for their ability to engage HSA using molecular docking. Digitoxin and BAC were also included because their binding regions on HSA are not well established. Our aim was to determine whether ligand binding could plausibly perturb albumin structure and its interaction network, thereby contributing to altered corona assembly. Docked poses were then evaluated by MD simulations under explicit solvent conditions at physiological temperature and ionic strength to assess the dynamic behavior and stability of each complex. These simulations provide insight into the persistence of ligand–protein interactions, the flexibility of the binding regions, and the capacity of ligand binding to reshape albumin-associated interactions over time.

#### Docking analysis:

The ligands were screened independently against the monomeric and dimeric forms of HSA. Docking simulations with the HSA monomer indicated that digitoxin exhibited the strongest predicted interaction, with a binding affinity of −10.24 kcal/mol. Phosphatidic acid, sphingomyelin, and CDP-choline followed with binding energies of −7.16, −6.79, and −6.50 kcal/mol, respectively (**Table S1**). In docking simulations performed with the HSA dimer, digitoxin again showed the strongest binding affinity (−9.88 kcal/mol), followed by CDP-choline (−7.51 kcal/mol). By comparison, BAC showed weaker predicted interactions with HSA, with docking scores of −5.86 and −5.32 kcal/mol for the monomeric and dimeric forms, respectively.

#### Site specific binding:

Analysis of the binding sites revealed a clear preference for specific HSA regions. A large subset of compounds, particularly phospholipids such as DMPC, DOPC, DPPC, PA, PE, PI, PS, phosphatidylcholine, and sphingomyelin, as well as BAC localized near Fatty Acid Binding site 1 (FA1). Sudlow’s site I was targeted by acetylcholine and cardiolipin, while choline and CDP-choline displayed alternative poses that also involved Site I. Digitoxin and LPC occupied positions between subdomains IA and IIIA, suggesting interaction competition between adjacent domains in mediating their binding (**Figures S1 and 1B**). Similarly, choline and CDP-choline exhibited mixed binding modes bridging FA1 and Site I. Overall, the site-specific analysis highlights FA1 as a dominant binding location with Site I and the IA/IIIA interface serving as additional, though less frequently targeted, sites.

The dimer binding analysis showed a similar trend, with FA1 emerging as the most frequently targeted site. Ligands including BAC, DMPC, DPPC, PA, PI, PS, phosphatidylcholine, and sphingomyelin localized close to FA1, predominantly on chain A. Site I was engaged by acetylcholine (chain B), CDP-choline (chain B), DOPC (chain B), LPC (chain B), and PE (chain B), indicating a broader affinity to this site by both small molecules and larger phospholipids. More specialized binding included digitoxin at the IA/IIIA interface (chain A) (**Figures S1 and 1B**), cardiolipin located between chains A and B, and choline displayed multiple poses, bridging FA1 and Site I across both chains. These findings suggest that FA1 remains a key anchor site in the dimer, while Site I plays an increasingly diverse role across both chains. Additionally, inter-chain and interface binding modes highlight the expanded range of potential interactions unique to the dimeric HSA system.

#### Binding affinity ranking using MD simulations:

The Coulombic and Lennard-Jones interaction energies over the 100 ns simulation times-series were calculated (**Figures S2 and S3**). The relative binding strengths of all ligands to HSA were assessed by the average short-range nonbonded interaction energies over the entire 100 ns production simulation. Combining the electrostatic Coulombic energy and the intermolecular potential of Lennard-Jones energy terms, provides a quantitative measure of binding strength to HSA. The rankings of ligand affinities to HSA based on interaction energy (**Figure S2a**) reveal a wide range of binding strengths to HSA. PS, and PA stand out as exemplary binders to HSA with average energies much lower than −500 kJ/mol. Acetylcholine, choline, and LPC have noticeably low interaction energies with HSA. This is consistent with their dissociation and detachment from their respective binding site starting positions throughout the simulation trajectory. (Note: digitoxin was not included in the MD simulations because its parameters were not available in the selected force field. Initial attempts to generate these parameters produced unreliable values).

Blind molecular docking resulted in ligands being grouped to different characteristic binding sites of HSA, notably Fatty Acid 1 (FA1) and Sudlow Site I. Time-series evolution of the total interaction energy (**Figure S2b-c**) of respective ligands to their sites revealed each ligand affinity to HSA over the 100 ns trajectory. Consistent with the previous rankings, FA1 site’s strongest ligands PS, and PA dominate the bottom of the time-series plot (**Figure S2b**), indicating higher binding affinity to the FA1 binding site. DPPC and DOPC are the lowest performing ligands with regards to this binding site. In the Sudlow’s Site I domain (**Figure S2c**), cardiolipin emerged as the strongest binder whereas smaller choline-based molecules demonstrated weak to negligible association. These results highlight distinct preferences between the two major HSA sites, with FA1 favoring phospholipids and Sudlow I accommodating larger species.

#### RMSD:

The Root Mean Square Deviation (RMSD) of a given ligand’s heavy molecules aligned to the protein backbone (**Figure S4**) provides a quantitative measure of the ligand’s stability during the 100 ns production run. Stable RMSD values indicate the preservation of the docked ligand pose and strong binding to HSA, whereas large or erratic deviations reflect rearrangement or detachment from their respective binding pocket. Smaller, mobile ligands such as acetylcholine, choline, CDP-choline, and LPC displayed rapid RMSD increases within the first few nanoseconds, followed by continued drift, consistent with dissociation into solvent or migration to some superficial site on the protein surface. Other ligands with moderate to strong affinity to HSA exhibit low and stable RMSD values throughout the simulation timesteps. Overall, the RMSD analysis supports the observed affinity ranking hierarchy, with ligands with the most favorable interaction energies maintaining the most stable conformations during the simulation. Stable RMSD time-series therefore serve as a structural indicator of tight, persistent binding between HSA and these ligands, while unstable RMSD’s indicate a lack of affinity between the ligand and HSA’s binding site.

#### RMSF:

The Root mean square fluctuation (RMSF) calculations describe the localized mobility of the ligand (**Figure S5**) and protein backbone (**Figure S6**). Acetylcholine and choline exhibited uniformly low RMSF values, reflecting both their small size and rigid binding poses. Cardiolipin, DMPC, DOPC, DPPC, and other phospholipids displayed sharply contrasting regions of rigidity and flexibility. This trend largely implies that their headgroups tend to form electrostatic interactions with HSA, whereas the lipid tails remain mobile within or partially unrestricted to move. The backbone RMSF of HSA remained similar for most residues, indicating that the overall protein structure was well maintained throughout all simulations. Residues located within or adjacent to the FA1 and Sudlow I binding domains exhibited the largest RMSF values. Systems containing strongly bound ligands, such as PS and PA showed locally lower RMSF around the binding pocket, consistent with stabilization caused by ligand interaction. In contrast, more weakly bound ligands, such as acetylcholine or LPC were associated with greater flexibility in these same regions, reflecting the absence of stabilizing ligand interactions.

#### Hydrogen bonding:

To characterize the polar interactions behind ligand affinity to HSA, the number of hydrogen bond counts characterized by short-range donor-acceptor contacts under 3.5 Å between HSA and each ligand were calculated over the simulation timesteps (**Figure S7**). DMPC, DOPC, PA and to a slightly lesser extent DPPC, PE, cardiolipin, and phosphatidylcholine are densely filled with blue and orange bars across most of the 100 ns simulation window, typically in the range of 2–6 hydrogen bonds. This is evidence of persistent, well-anchored binding, and polar structural features to facilitate such a number of hydrogen bonds. Acetylcholine, choline, CDP-choline, BAC, and LPC panels are mostly empty, with a few brief spikes, but almost no hydrogen bonds or contacts for the rest of the trajectory, consistent with rapid loss of specific interactions or detachment from the binding.

Overall, the interaction energy, RMSD, RMSF, and hydrogen bond analyses describe differences in binding strength, stability, and structural change between ligands. Ligands that exhibit strong binding show large total interaction energies, with high Coulombic and Lennard-Jones contributions. Their charged or polar headgroups likely form strong electrostatic and hydrogen bond interactions with residues in the FA1 pocket, while their hydrophobic tails are stabilized by van der Waals forces inside the nonpolar regions of the cavity. These systems also show low RMSD values and reduced RMSF, indicating stable complex formation and limited backbone motion.

A comparison between the monomer molecular docking and MD results reveals that there is a similar trend between the rankings of strong and weak ligand binding affinity to HSA, but the added complexity of interactions considered in MD simulations shifts the rankings of several ligands. Phospholipids such as PS and PA moved up in rank and became the strongest binders in the MD simulations, showing total interaction energies around −600 kJ/mol. These ligands likely gained additional interaction stabilization through the inclusion of longer-range electrostatic interactions of their phosphate heads and the dynamic stabilization of their hydrophobic tails, effects that docking does not capture. Other phospholipid molecules like cardiolipin, sphingomyelin, and PE also improved moderately in rank for the same reason. Docking assumes rigid structures and simplified scoring, so it misses solvent effects, entropic contributions, and conformational flexibility. MD includes these dynamic factors, revealing that some docked poses are unstable or that additional interactions form over time, leading to differences in apparent binding strength.

### Small molecules expand the plasma proteome coverage

To evaluate the capacity of our small-molecule panel to modulate protein corona composition and enhance proteomic depth, we incubated human plasma with individual compounds at two final concentrations, 1 and 10 mM, prior to exposure to 200 nm polystyrene NPs (**Figure S8**). We also evaluated five “molecular cocktails” (each consisting of two small-molecule binders) to determine whether synergistic engagement of multiple binding modes could further diversify protein recovery. Following NP incubation and isolation, the hard corona was processed using an optimized proteomics workflow^19^ for LC-MS/MS analysis (see the Methods section for details). For unbiased comparison between different conditions, each three replicates of plasma treated with a given small molecule condition were subjected to individual database searches.

To evaluate the reproducibility of protein quantification across experimental conditions, we calculated the coefficient of variation (CV) for each protein based on replicate measurements. The cumulative distribution of CV values is shown in **Figure S9**. Across all conditions, the majority of quantified proteins exhibited CV values below 30%, attesting to the reproducibility of the measurements.

**Figure 3.**
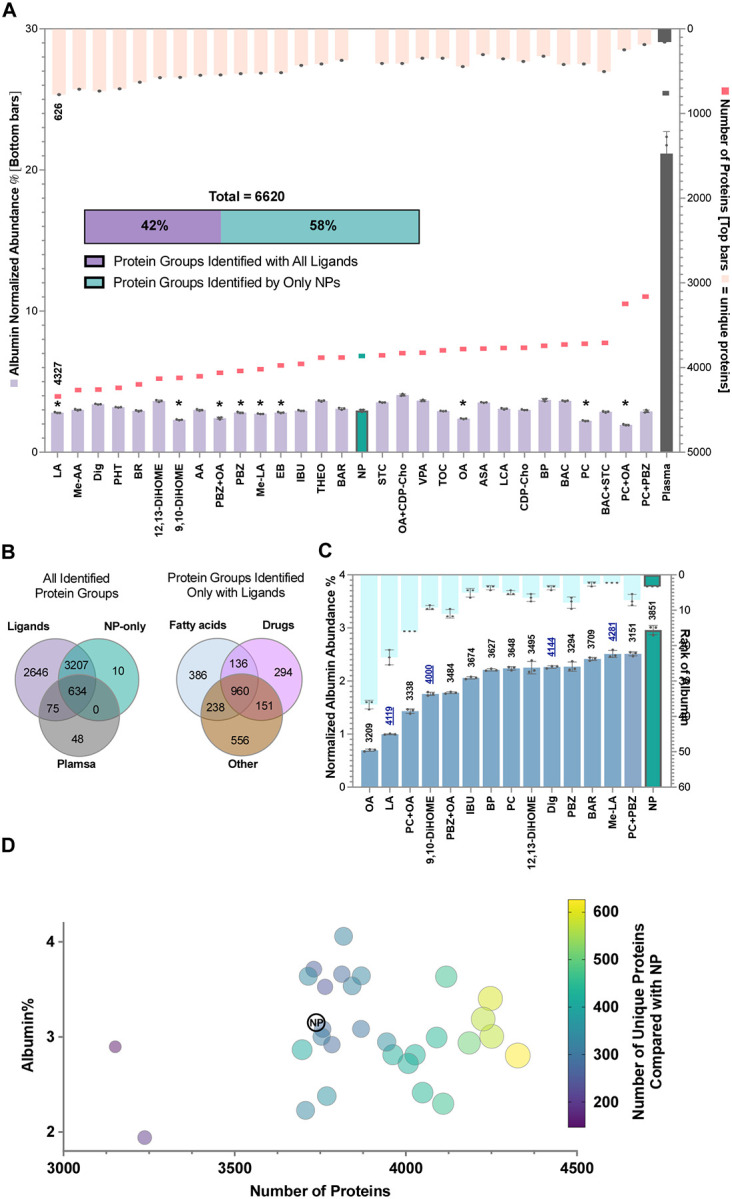
Small-molecule modulation of albumin abundance and protein coverage in the NP corona. (A) Albumin normalized abundance (left axis, bottom bars), number of identified protein groups across individual small-molecule conditions (right axis, coral squares), and number of unique protein groups quantified in each sample relative to the NP control (right axis, top bars). The stacked bar summarizes the total number of proteins identified across all conditions (6,508), partitioned into proteins uniquely identified in the presence of small molecules and those identified in untreated NPs (shown in green). Only the data for 1 mM concentration of small molecules or cocktails are shown in this graph. The bars indicated with asterisks have significantly lower albumin level compared with NP-only analyzed by one-way ANOVA multiple comparison. (B) Venn diagrams left: showing the overlap of proteins quantified in plasma, NP-only, and in the presence of ligands; right: showing the distribution of proteins quantified exclusively in small-molecule–treated coronas and absent from the NP-only control, grouped by FAs, drugs, and other molecules. (C) Comparison of albumin normalized abundance (left axis, bottom bars) and albumin rank within the corona proteome (right axis, top bars) for selected conditions (10 mM concentration of small molecules and cocktails) exhibiting statistically significant reduced albumin levels relative to NP. The numbers above the bars indicate the number of proteins identified under each condition. Dark blue underlined values indicate conditions with a higher number of identified proteins than NP-only. (D) Relationship between albumin abundance and proteome depth across small-molecule conditions.

Across all experimental conditions, we quantified a total of 6,620 unique proteins (Figure 3A, data deposited to the ProteomeXchange Consortium). Of the 757 proteins identified in human plasma in the absence of NPs, 123 were not recovered in the NP-only condition. The use of ligands recovered 75 of these proteins, leaving 48 proteins unique to plasma. In addition, NPs alone identified 3,851 proteins (58% of the total), and nearly all of these (99.7%) were also identified across the ligands panel (Figure 3B). Notably, the inclusion of our small-molecule panel significantly expanded the proteomic discovery space, enabling the identification of 2721 proteins (42% of the total dataset) that were not captured by untreated NPs alone (Figure 3A). These proteins, along with their corresponding families, were analyzed and mapped in **Figure S10**. From this pool of proteins uniquely identified in the presence of small molecules, 14%, 11%, and 20% of the proteins were identified by fatty acids, drugs, and the remaining molecules (others), respectively (Figure 3B, for classification of molecules, see Table 1). While the transition from neat plasma to untreated NPs reduced albumin abundance from 21% to 3% and increased the number of identified proteins from 757 to 3,851, our small-molecule strategy pushed these limits further. Several small molecules significantly reduced albumin levels compared with NPs alone at both 1 and 10 mM concentrations (see bars in Figure 3B with asterisk and the corresponding molecules in Figure 3C). At 1 mM, linoleic acid (LA) provided the highest coverage, yielding 4,327 proteins, of which 626 were unique to this condition. At 10 mM, this compound decreased the albumin to as low as 1% and increased its rank to 23 albeit with lower number of identified proteins compared to its lower concentration (Figure 3C).

### Small molecules expand the plasma proteome coverage by several mechanisms

Interestingly, our data suggest that while reduced albumin representation in the corona contributes to enhanced proteomic depth, it is clearly not the sole determinant. As shown in Figure 3D, the relationship between albumin level and the number of unique proteins identified is not strictly linear. Conditions with comparable albumin levels often yielded markedly different numbers of unique proteins, indicating that simply lowering albumin does not fully account for the observed increase in proteome coverage. Notably, the ligands associated with the highest numbers of unique proteins relative to the untreated NP condition (yellow-shaded circles on the right side of Figure 3D) span a broad range of albumin levels and do not correspond to the conditions that produced the lowest albumin abundance. These findings suggest that the gain in protein identifications arises not only from partial displacement of albumin, but also from ligand-dependent reorganization of protein–protein and protein–NP interactions. Such effects may alter which proteins remain associated with albumin, which are released into solution, and which become accessible for NP capture, thereby reshaping corona composition in ways that enhance detection of lower-abundance proteins. This implies that the identity of proteins incorporated into the corona, and the resulting proteomic depth, are governed by more complex molecular mechanisms than albumin reduction alone.

We hypothesize that three primary factors dictate the outcome of ligand-mediated corona remodeling consisting of (1) competitive occupancy and displacement, (2) allosteric conformational shifts, and (3) electrostatic surface remodeling. Because a significant fraction of plasma albumin is endogenously bound to FAs, the introduction of exogenous ligands creates a competitive environment. Ligands that overlap with FA-binding sites can displace endogenous cargo, altering the protein’s native state and its subsequent interaction with the NP surface; this forms the basis of the competitive occupancy and displacement hypothesis. In addition, albumin’s inherent flexibility allows ligand binding at one site to trigger long-range structural rearrangements. These shifts can hide or expose hydrophobic patches that are critical for NP adsorption. Finally, ligand binding can significantly alter the surface charge distribution of albumin. For example, structural comparison of phenylbutazone-bound albumin with its apo form reveals a pronounced increase in negative electrostatic surface potential (Figure 2). Given the partially negative surface of the carboxylated polystyrene NPs, this heightened negativity likely induces electrostatic repulsion, thereby disfavoring albumin adsorption and freeing surface area for lower-abundance species.

In contrast to single-ligand treatments, certain molecular cocktails showed divergent results. The combination of OA and PBZ (at 1 mM) successfully decreased albumin abundance and increased the proteomics depth relative to OA alone, suggesting a synergistic effect. Conversely, co-treatment with OA and PC reduced the number of quantified proteins, likely due to antagonistic binding effects or localized precipitation, highlighting the importance of precise ligand selection in targeting multiple sites of albumin.

Collectively, our findings demonstrate that structurally diverse small molecules can be strategically employed to reshape the NP protein corona, with reduced albumin incorporation representing only one component of a broader proteome-level effect. This behavior is not merely a function of ligand binding affinity; rather, it is dictated by a tripartite interplay among site-specific occupancy, allosteric structural rearrangements, and resulting surface property alterations, together with likely perturbations to interactions involving albumin, other highly abundant plasma proteins, and the surrounding proteomic network. Consequently, we establish that distinct molecular classes (e.g., long-chain FAs versus heterocyclic drugs) govern the ultimate composition of the NP corona through divergent biophysical pathways. Importantly, the resulting increase in protein identifications cannot be explained solely by decreased albumin levels. Instead, ligand-driven reorganization of protein–protein and protein–NP interactions appears to alter the accessibility of proteins across the plasma proteome, thereby facilitating the capture of species that are otherwise underrepresented or not detected in the untreated corona. By altering the conformational and adsorption landscape of abundant plasma proteins, these ligands influence not only the quantity of albumin recruited but also the breadth and composition of the sub-dominant proteome occupying the NP surface. Ultimately, this ligand-directed restructuring provides a tunable mechanism to rationally engineer protein corona composition, enhancing detection of lower-abundance proteins, as will be highlighted below, while also offering critical insights for controlling NP biodistribution and therapeutic efficacy.

### Small molecules extend the limit of detection

**Figure 4.**
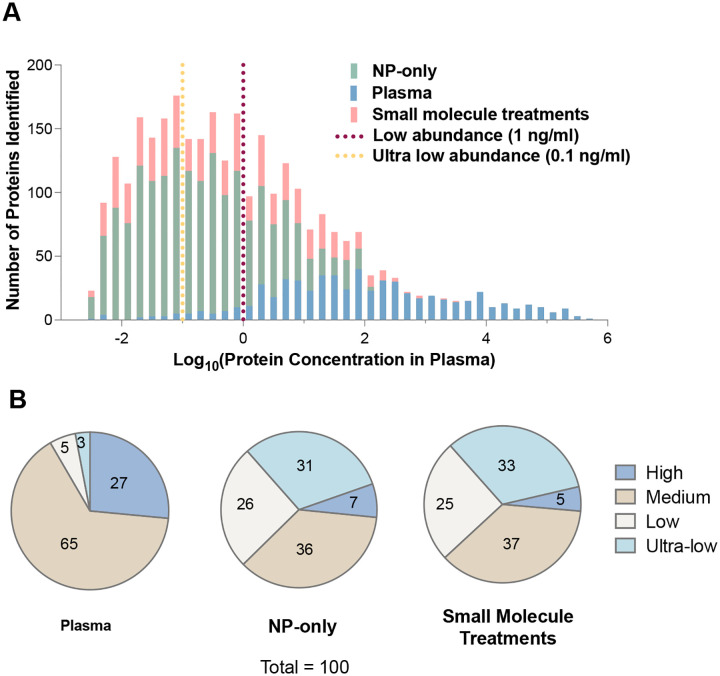
The representation of low-abundance proteins in the dataset. (A) Distribution of detected proteins across plasma concentration ranges based on reported plasma abundance values. Histograms compare proteins identified in plasma, NP-only corona, and small-molecule–treated samples. Vertical dashed lines indicate the thresholds for low-abundance (1 ng/mL) and ultra-low-abundance (0.1 ng/mL) proteins. (B) Proportion of proteins detected in each abundance class (high: >1000 ng/ml, medium: 1–1000 ng/ml, low: <1 ng/ml, and ultra-low: <0.1 ng/ml) for plasma, NP-only corona, and small-molecule–treated samples.

The plasma proteome spans an exceptionally wide dynamic range of over 10 orders of magnitude, from highly abundant proteins such as albumin and apolipoproteins present at mg/mL concentrations to low-abundance signaling molecules such as interleukins in the pg/mL range. To evaluate the clinical and biological relevance of our enhanced proteomics depth, we mapped the quantified protein groups to the Human Protein Atlas (HPA) blood proteome database concentration tiers. We categorized the captured proteome into high-, low-, and ultra-low-abundance cohorts (Figures 4 **and S11A-D**). This analysis confirms that our small-molecule modulation strategy does not simply increase protein yield or act through a straightforward reduction in HSA levels. Instead, it appears to reshape the plasma proteome landscape in a manner that increases NP access to lower-abundance proteins. As a result, the detectable proteome shifts toward low- and ultra-low-abundance species, many of which are not observed in standard NP-only workflows. Consequently, this strategy provides a high-resolution snapshot of the plasma environment that includes rare regulatory and leakage proteins that would otherwise remain masked in the untreated condition. Notably, the normalized abundance of proteins detected in plasma showed a positive correlation with their reported plasma concentrations in HPA, further supporting the quantitative consistency and biological relevance of the dataset (**Figure S12**).

### Small Molecules Reshuffle the Top Proteins within the Corona

**Figure 5.**
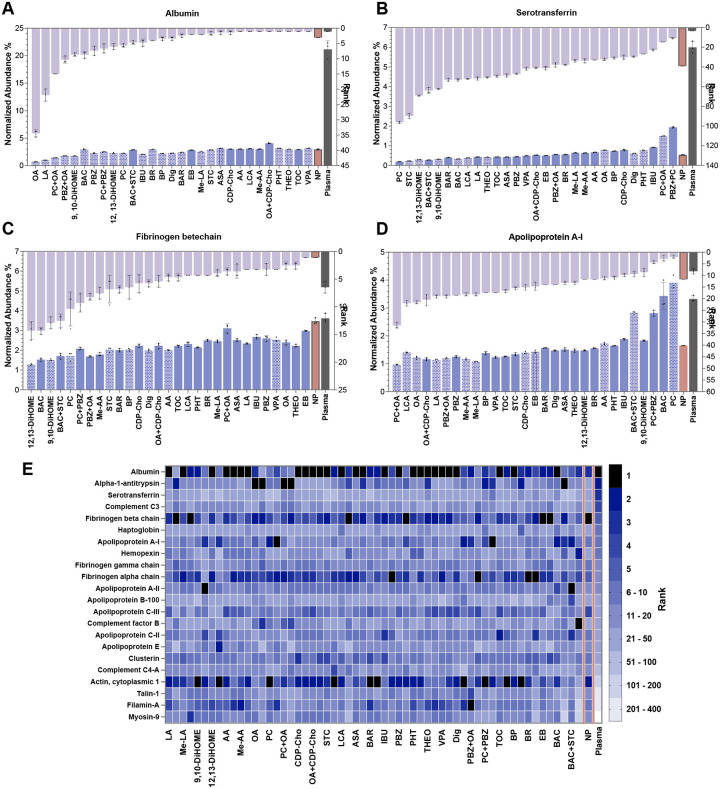
Distribution of the most abundant proteins across conditions. (A), (B), (C), and (D) Rank and relative abundance of the high-abundance plasma proteins albumin, serotransferrin, fibrinogen beta chain, and Apolipoprotein AI across plasma, untreated NP coronas, and small-molecule–treated coronas. Only the best performing concentration is shown for each compound. Patterned bars indicate the 10 mM samples. (E) Heatmap showing the most abundant proteins identified in each sample, with numbers indicating their rank in abundance within each condition. Columns are grouped by compound, with each pair representing the 1 mM condition followed by the corresponding 10 mM condition.

While attenuation of albumin’s interactions with the proteome and NPs is an important feature of our approach, the effects of small-molecule interference extend across the broader hierarchy of abundant plasma proteins. In the untreated NP corona, several highly abundant proteins, including fibrinogen and serotransferrin, remain strongly represented on the NP surface, thereby limiting access of lower-abundance species. Our results show that targeted addition of small molecules induces a secondary reorganization of these top-tier proteins, reducing their surface representation beyond that observed with NPs alone (Figures 5 and **S13**). These findings support a model in which small molecules do not act solely by diminishing albumin incorporation, but more broadly reshape competitive protein–protein and protein–NP interactions, thereby increasing access of lower-abundance proteins to the NP surface.

For instance, serotransferrin (~6% in neat plasma) was reduced to rank 38 in the untreated corona, but the addition of PC, STC, 9,10-DiHOME and 12,13-DiHOME further reduced its representation on the NP surface, shifting it to ranks as low as 94 (Figure 5B). Similarly, fibrinogen β chain, which remained the most abundant protein in the untreated corona (rank 1), was markedly displaced by 9,10-DiHOME, falling to rank 12 (Figure 5C). These shifts indicate that the small molecules do not merely alter albumin binding, but more broadly reorganize the competitive adsorption landscape at the NP surface. By perturbing protein–protein and protein–NP interactions, they appear to destabilize secondary adsorption networks that otherwise favor retention of major abundant proteins, thereby increasing access for lower-abundance species. To visualize this reorganization, we mapped the most abundant proteins recruited under various conditions (Figure 5E). Our results indicate that small molecules can systematically deconstruct the dominance of the top-tier plasma proteome, effectively clearing surface real estate for the detection of low-abundance proteins.

### Functional Classification of the Remodeled Corona

**Figure 6.**
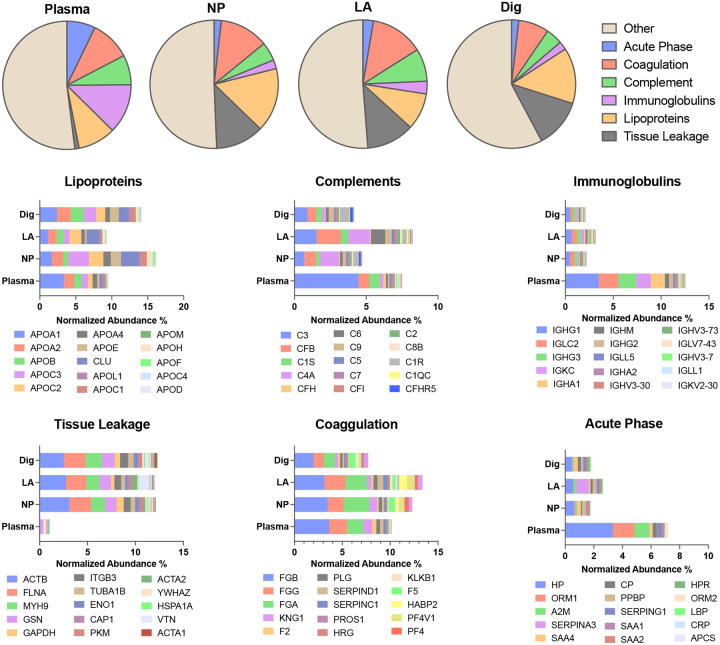
Small-molecule modulation reshapes the functional composition of the plasma protein corona. Pie charts show the overall distribution of major protein classes in plasma, NP-only, and representative small-molecule conditions (LA and Dig), including acute phase, coagulation, complement, immunoglobulins, lipoproteins, tissue leakage, and other proteins. Stacked bar plots below highlight the relative contributions of individual proteins within each class.

To determine how small-molecule modulation reshapes the biological landscape of the protein corona, we categorized the identified proteome into six functional classes based on gene annotations: lipoproteins, immunoglobulins, complement proteins, acute-phase proteins, coagulation factors, and tissue-leakage proteins.^52^ These classes consistently represent approximately 50% of the total protein mass across all conditions (Figures 6, **S14**, **S15**, and **S16**). However, the relative distribution of these classes varies significantly between neat plasma and the NP corona, revealing a profound selection bias induced by both the NP surface and our small-molecule panel.

#### Coverage of tissue leakage proteins.

The most striking transition occurred within the tissue-leakage category. While these proteins constitute a negligible fraction (~1%) of the neat plasma proteome, their representation increases more than ten-fold upon corona formation, reaching 10–12% in NP and small-molecule-treated samples. This class is notably dominated by intracellular markers which serve as direct indicators of cellular distress or organ-specific pathology. The ability of our platform to preferentially capture this “hidden” leakage proteome highlights the transformative power of small-molecule modulation in further uncovering tissue-specific markers by NPs’ alone that are otherwise masked by high-abundance circulatory proteins (Figures 6, **S15**, and **S16**).

#### Suppression of high-abundance analytical background.

A critical requirement for deep proteome profiling is the reduction of dominant plasma protein classes that can saturate MS spectra and limit detection of lower-abundance species (Figures 6, S15, and S16). We observed a systematic reduction in these highly abundant background proteins. For example, immunoglobulins decreased from ~12% in plasma to 2–5% in the corona. Small-molecule addition effectively reduced the surface representation of these highly abundant antibodies (e.g., IGHG1), thereby freeing NP binding capacity for lower-abundance species. Similarly, acute-phase proteins, including dominant inflammatory markers such as A2M and HP, were reduced from a combined ~14% in plasma to approximately 6–8% across corona conditions.

#### Tunable selectivity via lipoprotein and complements modulation.

Lipoproteins exhibited the highest variability, suggesting a customizable diagnostic surface. Specific conditions, such as LA, significantly reduced the abundance of markers like APOA1 compared with NPs, whereas Dig increased the abundance of the same protein. This demonstrates that specific ligands can tune the NP surface to selectively recruit or exclude lipid-transporting complexes, providing a tunable handle to tailor the corona for specific disease states, such as metabolic or cardiovascular disorders. Interestingly, for complement proteins, these small molecules showed the opposite trend. LA increased their relative abundance, while Dig maintained them at levels comparable to those observed with NPs alone (Figures 6).

By strategically deconstructing the dominance of the high-abundance plasma proteome, our small-molecule approach clears the analytical space necessary to detect the rare, tissue-derived signals required for next-generation liquid biopsies and early-stage disease screening.

### Remodeled Protein Corona Improves Detection of FDA-Approved Plasma Biomarkers

**Figure 7.**
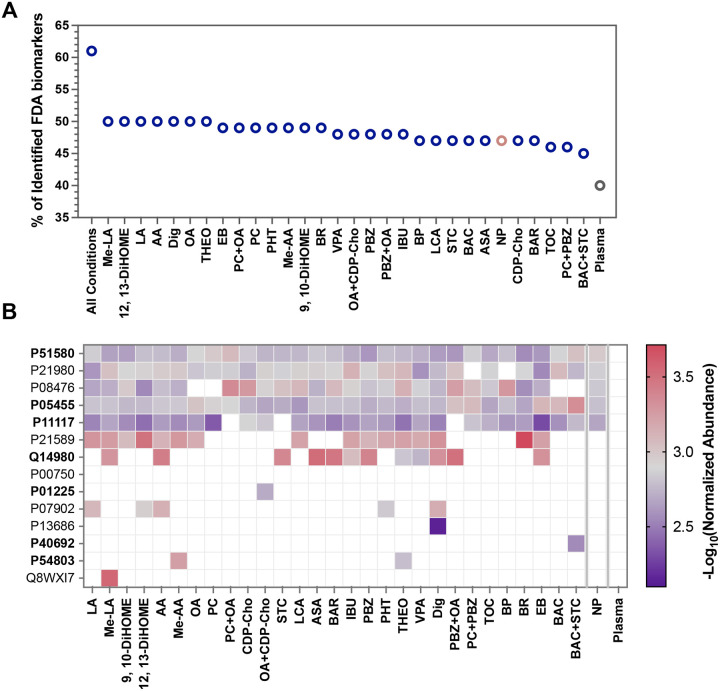
Identification of low-abundance FDA-approved biomarkers across experimental conditions. (A) Percentage of FDA-approved biomarkers identified in the NP corona under different small-molecule conditions. Each point represents the best-performing concentration for each compound, indicating the fraction of the total FDA biomarker panel detected in that sample. The “All conditions” point represents the combined identification across all datasets, while plasma is shown for comparison. Most conditions detect approximately half of the known FDA biomarkers, demonstrating broad coverage of clinically relevant proteins. (B) Heatmap showing the normalized abundance of selected low-abundance FDA-approved biomarkers across experimental conditions. Rows represent individual proteins (UniProt IDs) and columns correspond to different conditions. The color scale represents the −log_10_ of normalized abundance values, highlighting relative enrichment of these biomarkers across the tested NP corona conditions. White squares indicate proteins not detected in a given condition. Only the best performing concentration is shown for each compound

To quantify the clinical relevance of our enhanced proteomic depth, two reference datasets of 240 clinically relevant proteins were compiled from published resources, including FDA-approved biomarker proteins and proteins associated with FDA-approved diagnostic assays obtained from MRMAssayDB.^48, 49^ Remarkably, 147 (61%) of these clinical markers were detected in at least one experimental condition (Figure 7A). The proportion of identified biomarkers expanded progressively from 40% in neat plasma to 47% in the untreated NP corona, reaching a maximum of 50% following targeted small-molecule modulation. While 84 biomarkers were ubiquitously detected across all conditions, a significant subset of “non-shared” markers was revealed only through NP-mediated enrichment (**Figure S17**).

To assess the analytical sensitivity required for early-stage diagnostics, we analyzed these non-ubiquitous biomarkers using reported plasma concentrations from the HPA. Our small-molecule strategy successfully exposed 12 low-abundance biomarkers with absolute concentrations below 1 ng/mL, half of which were classified as ultra-low-abundance (0.1 ng/mL) (Figure 6B). Crucially, none of these 12 high-value markers were detectable in neat plasma analysis. While the untreated NP corona captured four of these proteins, the remaining eight biomarkers, including three in the ultra-low-abundance category, were identified exclusively following small-molecule modulation. Notably: i) Mucin-16 (CA-125) which is detected uniquely in the presence of 1 mM Me-LA (CA-125 is the gold-standard biomarker for monitoring ovarian cancer recurrence); ii) TRACP-5b which is identified with 1 mM digitoxin (this protein is a highly specific marker for bone resorption and osteoclast activity in metabolic bone diseases). Two ultra-low abundance biomarkers, DNA mismatch repair protein MLH1 and galactocerebrosidase were detected in BAC+STC mix, and Me-AA and THEO respectively. MLH1 is a nuclear protein involved in DNA mismatch repair and is clinically associated with Lynch syndrome and colorectal cancer diagnostics, while galactocerebrosidase is a lysosomal enzyme involved in galactolipid metabolism and is used in screening for Krabbe disease.

Together, these findings demonstrate that small-molecule remodeling of the NP corona does not merely increase the number of identified proteins; it enriches clinically validated, low-abundance species that remain invisible to conventional plasma proteomics. This capability significantly extends the detectable biomarker coverage, providing a robust framework for high-sensitivity liquid biopsies.

### Remodeled Corona Expands Mapping of the Low-Abundance Cytokine Repertoire

Circulating cytokines are critical molecular sentinels of systemic immune homeostasis and pathology; however, their clinical utility in discovery proteomics is restricted by their ultra-low concentrations, which typically fall below 1 ng/mL.^9^ To assess whether ligand-mediated corona remodeling could bridge this sensitivity gap, we benchmarked our platform’s cytokine coverage against a curated library of 179 secreted immune mediators derived from the HPA and recent literature.^50^

**Figure 8.**
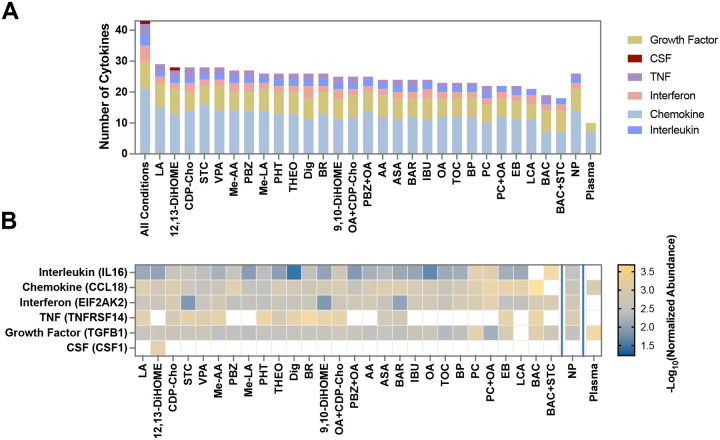
Cytokine family distribution across different experiment conditions. (A) Each bar represents a distinct treatment condition, and the stacked segments indicate the absolute number of cytokines assigned to each of six immunological families: Interleukins, Chemokines, Interferons, TNF superfamily, Colony-Stimulating Factors (CSF), and Growth Factors (pink). (B) Heatmap showing the normalized abundance of selected cytokines IL16, CCL18, EIF2AK2, TNFRSF14, CSF1, and TGFB1 across the experimental conditions. Rows represent selected cytokines and columns correspond to different conditions. The color scale represents the −log_10_ of normalized abundance values, highlighting relative enrichment of these cytokines across the tested NP corona conditions. White squares indicate proteins not detected in a given condition. Only the best performing concentration is shown for each compound.

As shown in Figure 8, our multi-ligand corona platform quantified 39 unique cytokines (**Figure S18**), representing a 3.9-fold expansion over neat plasma (10 cytokines) and a 1.7-fold increase over untreated NP controls (26 cytokines). While individual small-molecule modifiers yielded a maximum per-condition coverage of 29 cytokines, the intersectional analysis revealed profound heterogeneity: only 4 cytokines were common to all conditions including plasma. This distinct selectivity underscores a combinatorial advantage; wherein chemically diverse ligands confer complementary enrichment profiles that collectively broaden the window of immune surveillance.

Compositional analysis of the enriched “cytokinome” showed a predominance of chemokines (49%), with the remaining identifications spanning interleukins, growth factors, interferons, and TNF superfamily members. Notably, the relative representation of these families shifted according to the ligand used, suggesting that ligand modulation can be tuned to favor specific functional classes of signaling molecules.

To benchmark our sensitivity against established clinical thresholds, we mapped our findings to HPA-reported plasma concentrations. Of the 43 cytokines detected, 25.6% were classified as low-abundance (<1 ng/mL) and 14.0% as ultra-low-abundance (<0.1 ng/mL). High-priority mediators of inflammatory signaling were disproportionately represented in these lower tiers; for instance, 67% of detected TNF superfamily members and 56% of growth factors were exclusively identified in the low-abundance range.

In stark contrast, neat plasma analysis failed to recover any representatives from the interferon, TNF, or interleukin families, identifying only medium-to-high abundance chemokines and growth factors (≥1 ng/mL). These results demonstrate that ligand-induced corona remodeling systematically overcomes the dynamic range bottleneck, enabling the selective concentration of structurally and functionally diverse immune mediators that are otherwise lost to the albumin-dominated background.

### Enhanced Surveillance of the Pan-Disease Blood Proteome

Beyond canonical immune mediators, the trace-level concentrations of clinically actionable disease biomarkers pose a formidable challenge for conventional discovery proteomics. To evaluate the capacity of NP corona-based profiling to systematically expand the accessible biomarker space, we cross-referenced our enriched proteins against a curated pan-disease blood proteome atlas comprising 1,165 proteins significantly dysregulated across 59 clinical conditions (HPA blood PEA atlas, p-adjusted < 0.05 vs. healthy controls).^48^

Our multi-compound platform identified 496 of these unique disease biomarkers—a 3.8-fold expansion over neat plasma (130 biomarkers) and a 1.5-fold increase over untreated NP controls (330 biomarkers). At the individual-condition level, ligand modulation resulted in capturing up to 340 biomarkers, more than doubling the depth of neat plasma analysis. Comparative analysis across conditions revealed profound enrichment heterogeneity; only 15% of biomarkers were common to all conditions, whereas the vast majority (421 proteins, 85%) exhibited condition-specific enrichment. This high degree of orthogonality confirms the combinatorial necessity of a multi-ligand strategy for broad-spectrum disease surveillance (**Figure S19**).

The identified biomarkers spanned a wide clinical spectrum. Using a primary-class assignment based on the most significant disease association per protein (lowest adjusted p-value vs. healthy controls), the distribution was dominated by markers of infection (32.1%), followed by autoimmune (30.6%), oncological (17.7%), pediatric (11.9%), and metabolic (6.0%) pathologies (**Figure S19**). Notably, nearly all detected biomarkers were significantly dysregulated across multiple disease classes, reflecting the highly pleiotropic nature of blood-borne disease mediators. When mapped to established concentration thresholds in the HPA, 27.1% of identified biomarkers fell within the low-abundance regime (<1 ng/mL) and 9.4% resided in the ultra-low-abundance tier (<0.1 ng/mL). Crucially, high-priority translational targets were disproportionately represented in these lower tiers; 35% of oncological and 27% of infectious biomarkers were classified as low-abundance, reflecting the characteristic scarcity of tumor-derived and host-response mediators in circulation.

In contrast, neat plasma analysis predominantly recovered medium-to-high abundance proteins (≥1 ng/mL), with only 1.6% of identified biomarkers falling into the low-abundance range. Notably, biomarkers primarily associated with autoimmune pathologies, proteins whose most significant dysregulation was observed in autoimmune disease contexts, were undetectable in neat plasma but were consistently recovered via ligand-modulated corona enrichment. This selective recovery underscores the capacity of the NP platform to access low-abundance, disease-specific signals that are below the detection threshold of conventional plasma proteomics. While this study utilized healthy donor plasma to establish a baseline, the robust detection of these “latent” biomarkers—proteins associated with disease but present at trace levels in health—suggests that this platform could offer unprecedented sensitivity for detecting early-stage pathophysiological shifts. These findings underscore the capacity of rationally remodeled coronas to concentrate disease-relevant proteins across a broader clinical spectrum than previously accessible.

## Supplementary Material

Supplementary Files

This is a list of supplementary files associated with this preprint. Click to download.

- SI41426Final.pdf

## Figures and Tables

**Table 1.** Details of the employed small molecules in this study and their interactions with albumin.

Name	Interaction with HSA
**Drugs**	
Theophylline (THEO)	Binds near the Trp214 in domain IIA, as indicated by synchronous fluorescence spectroscopy of the albumin-THEO complex.^26^
Barbital (BAR)	Binds within subdomain IIA of albumin, based on spectroscopic analyses conducted with bovine serum albumin (BSA).^27^
Phenytoin (PHT)	It is characterized by high plasma protein binding, with approximately 90% of the drug primarily bound to albumin and a smaller fraction associated with globulins and lipoproteins.^28^ Phenytoin interacts with multiple hydrophobic pockets on albumin, potentially including Sudlow site I, though the exact binding locale remains uncertain.^29, 30^
Acetylsalicylic acid (ASA)	Fluorescence quenching studies and molecular modeling have demonstrated that ASA primarily binds to albumin within subdomain IIA (site I).^31^ Additionally, aspirin can acetylate multiple lysine residues on albumin in a concentration-dependent manner, modifying the protein’s surface charge and impacting its binding behavior.^32^
Phenylbutazone (PBZ)	Phenylbutazone primarily interacts with domain IIA (Sudlow site I), while ibuprofen binds to domain IIIA (Sudlow site II).^24, 33^
ibuprofen (IBU)	Circular dichroism (CD) analyses of human serum albumin-IBU complexes reveal an increase in α-helix content and a decrease in β-sheet structures, consistent with ligand-mediated stabilization of the native fold and reduced exposure of hydrophobic patches.^34^
Sodium Valproate (VPA)	Binds to both site I and site II on albumin, with a higher affinity for site II.^35,34^
Digitoxin (Dig)	Binds to a distinct site on albumin, separate from the primary drug-binding regions at site I and site II.^36^
**Fatty Acids**	
Oleic acid (OA)	Faey acids are the primary endogenous ligands of serum albumin. Binding occurs within long, hydrophobic channels, with entrances stabilized by polar, predominantly basic amino acid side chains. *In vivo*, most FAs associated with HSA are unsaturated. Mono-unsaturated oleic acid (OA), di-unsaturated linoleic acid (LA), poly-unsaturated arachidonic acid (AA) are the most common types associated with serum albumin.^39^ These molecules engage multiple FA binding sites, with the highest affinity reported for the FA5 site in IIIA domain. In contrast to the largely localized perturbations induced by many drug ligands, long-chain FAs trigger allosteric conformational shifts that affect regions beyond the immediate binding site.^24, 35, 40^
Linoleic acid (LA)	
Arachidonic acid (AA)	
**Others**	
**Bile Acids**	
Lithocholic acid (LCA)	Bile acids can bind to both albumin and lipoproteins. Notably, their affinity for lipoproteins decreases as the polarity of the steroid nucleus increases.^37^ Sudlow site I is the primary binding site for bile acids.^38^
Sodium taurocholate (STC)	
**Vitamins**	
Tocopherols (TOCO)	Interacts with albumin mainly via hydrophobic interactions. Site marker competition studies have further identified site II as its preferred binding region.^41^
**Surfactant**	
Benzalkonium chloride (BAC)	MD simulations predict that BAC predominantly binds to Sudlow site II of albumin.^42^ Studies on BSA have demonstrated that BAC derivaJves with longer alkyl chains exhibit stronger binding affinities compared to those with shorter chains.^43^
**Endogenous and Synthesized Dyes**	
Bilirubin (BR)	This endogenous pigment produced during heme catabolism, is transported in the circulation primarily bound to HSA. While the precise bilirubin-binding site on HSA is debated, crystallographic studies have identified an L-shaped bilirubin-binding pocket within subdomain IB.^44^
Evans blue (EB)	Binds strongly to serum albumin both *in vivo* and *in vitro* with little dye remaining unbound. It has therefore been widely used as a protein tracer in clinical and diagnostic applications.^45^
**Hydrophobic Aromatic Compounds**	
Benzophenone(BP)	Binds human albumin based on spectroscopic and competition studies.^46^ Displacement assays with oleic acid (a known site I binder) and flufenamic acid (a site II binder) confirmed that BP predominantly binds at Sudlow site II.^46^

## Data Availability

Proteomic data have been deposited to the ProteomeXchange Consortium (https://www.proteomexchange.org/) via the MassIVE partner repository (https://massive.ucsd.edu/) with MassIVE data set identifier MSV000101259 and ProteomeXchange identifier PXD076238.
